# Supplementary material for: Are carbon emissions trading and green financial instruments synergistic? -Comprehensive quantitative research based on content analysis
Source: PLoS One. 2024 Mar 7;19(3):e0298601. doi: 10.1371/journal.pone.0298601 (PMC10919601; doi:10.1371/journal.pone.0298601)
Supplement: S1 Data — (ZIP) [file pone.0298601.s001.zip › Data/地级市Z_F工作报告.zip!/╔╜╬≈/╤⌠╚¬╩╨/└┤╫╘╣1⁄2╓┌║┼í╛┬φ┐╦╩2╛▌═°í┐_2019.docx]

各位代表：

现在，我代表市人民政府向大会报告工作，请予审议，并请市政协委员和列席人员提出意见。

一、2018年工作回顾

2018年是全面贯彻党的十九大精神的开局之年，是改革开放40周年。一年来，面对严峻复杂的国际国内形势和艰巨繁重的改革发展稳定任务，在市委的坚强领导下，全市上下坚持以习近平新时代中国特色社会主义思想为指引，坚决贯彻党的十九大精神和习近平总书记视察山西重要讲话精神，认真落实省委、市委各项决策部署，按照聚力六大突破、实现转型崛起总体要求和“123335”工作部署，全力抓好稳增长、促改革、调结构、惠民生、防风险各项工作，经济保持稳中有进、稳中向好的态势，各项社会事业发展取得了可喜的成绩。

全市地区生产总值增长6.7%，与全省同步；规模以上工业增加值逐季回升，全年增长5%；一般公共预算收入增长15.2%，连续两年保持15%以上较高增速；社会消费品零售总额增长7.2%；城乡居民人均可支配收入分别增长6.4%和8.6%，农村居民收入增速持续快于城镇居民；城镇登记失业率为3.08%，低于省定4.2%的控制目标；居民消费价格上涨1.8%，控制在3%的目标以内。空气质量优良天数比例虽未完成年度目标任务，但已取得明显改善，其余约束性指标都较好地完成了年度目标。

一年来，我们主要做了以下工作：

对标“示范区、排头兵、新高地”三大目标，转型发展迈出新步伐。经济结构持续优化。从产业结构看，三次产业占比为1.5∶47.0∶51.5，三产增速超过二产1.9个百分点，对GDP增长的贡献率达到57.1%。从工业结构看，煤炭占比持续下降，非煤产业占比提高2.9个百分点。从投资结构看，制造业投资增长87%，工业技改投资增长25.4%，为实现工业结构反转奠定了基础。新旧动能加速转换。裕光煤电、平定化工乙二醇等为代表的煤电化产业链条不断延伸，华越、华鑫等为引领的煤机制造产业向高端化、智能化、国际化发展，百度云计算、自动驾驶车路协同示范区等智能物联网应用基地建设项目顺利推进，现代物流、新材料、文旅康养产业加快发展，多元产业体系正在形成。新兴产业工业增加值增长5.9%，战略性新兴产业增加值增长7.4%，新一代信息技术服务业营业收入增长59.3%，旅游总收入增长26.5%。新登记各类市场主体15878户，增长14%；新增“小升规”企业22户、高新技术企业19家。能源革命成效明显。持续推动煤炭“减优绿”，关闭矿井3座，退出产能205万吨，先进产能占比达到68.7%，高于全省平均水平。光伏领跑者计划一期项目并网发电，新能源电力装机容量占比达到35.2%，全省第二。对外开放不断深入。山西（阳泉）国际陆港运营良好，“阳泉-天津港-佛山”公铁海联运班列开行，天津自贸区进口直营中心开业。深化招商引资工作，举办招商推介会44次，签约项目138个，当年开工率达到47.8%。在省委“三大目标”的正确导航下，发展的“含金量”“含新量”“含绿量”不断提升！

加快补齐“三块短板”，协同发展取得新进展。金融支撑更加坚实，全年各项存款余额增长6.3%，贷款余额增长16.8%，存贷比65.46%，全省排名第二。设立中小微企业信保基金，为300多户中小微企业贷款融资9.6亿元，应急周转保障资金累计使用16亿元，7家企业在山西股权交易中心“晋兴板”挂牌。创新动力更加强劲，设立高新技术产业股权投资基金、科技创新创业贷款风险补偿资金，发放各类科技奖补资金2100万元，建成各级各类众创空间、孵化器22家，全市技术合同交易额完成5.6亿元，万人有效发明专利拥有量达到1.38件。民营经济更加活跃，出台并实施了促进民营经济发展“1+6”行动方案，成立“泉民投”公司。全市新认定10家省级民营科技企业、8户省级“专精特新”中小企业，为中小企业发放各类奖励扶持资金1200万元。民营经济营业收入增长8.3%，税收增长13.2%。高质量发展的短板正在补齐加长，基础更加坚实稳固！

坚决打好“三场硬仗”，全面小康基础得到新巩固。重大风险总体可控，积极处置不良贷款，严防严打非法集资等经济犯罪，全市不良贷款余额和占比分别下降12.2%、0.9%。严格规范各类政府举债融资行为，加强政府隐性债务管理，多措并举偿还政府存量债务2.84亿元。精准脱贫决战决胜，完成了最后32个贫困村、3300名贫困人口脱贫任务。污染防治力度空前，蓝天、碧水、净土三大保卫战扎实推进，中央、省委环保督察和生态环境部约谈、强化督查问题全面整改，大气环境质量不断好转。空气质量综合指数下降9.6%，PM2.5等六项污染物平均浓度全部下降，省控以上地表水优良断面和劣V类断面比例均完成年度目标任务，全年完成各类营造林6.72万亩。三大攻坚战取得了阶段性胜利！

坚持深化改革不动摇，高质量发展再添新动力。国企改革全面突破，坚持彻底改、依法改、平稳改的原则，将市属74户国有企业全部纳入改革范围。21户僵尸企业一次性进入破产司法程序或者实施兼并重组，历史遗留的老大难问题取得重大突破。31户经营性企业完成了审计评估，全面推进深度混改。18户特殊功能类企业完善了法人治理结构和市场化经营机制，4户留守机构实现重组。“三供一业”移交工作走在全省前列，创造了阳泉模式。开发区改革全面提质，管理体制机制基本理顺，“三化三制”改革深入推进，阳泉经济技术开发区社会事务剥离工作顺利完成。平定经济技术开发区创新体制机制，与阳煤集团合作推进管运分离改革。全市开发区投资强度、产出强度、税收强度等指标超额完成省定任务。农村集体产权制度改革全面铺开，作为全国改革试点市，全市88%村(组)完成经营性资产折股量化，64%村(组)成立了集体经济组织，集体经济空壳村全部消除。此外，电力体制、医疗体制、综合行政执法体制等各项改革都取得了明显成效。制约发展的体制性障碍逐渐破除，一些具有阳泉特色的改革事项正在进入全省第一方阵！

大力实施乡村振兴战略，城乡融合发展呈现新气象。编制完成城乡融合发展总体规划和6个专项规划。现代特色高效农业蓬勃发展，有机旱作农业、城郊农业、休闲农业等不断壮大，粮食生产再获丰收。美丽乡村展现新风貌，盂县王炭咀、平定娘子关、郊区后洼3个村新晋美丽宜居示范村行列，王炭咀村荣获中国美丽休闲乡村称号。城乡统筹步伐加快，平阳街一期、中环快速路西北段建成通车，市蔬菜果品批发市场搬迁新址运营，“四好农村路”建设任务圆满完成。阳大铁路、综合交通客运枢纽、太行山一号旅游公路、桃河湿地公园等项目顺利推进。“五城联创”成效显著。创建国家卫生城市取得阶段性成效，实施创卫工程8176项，拆除违建15.2万平米，新建改造公厕188座。集中开展拆违治乱、市容市貌、垃圾清运、食品卫生等专项整治，形成了全民参与、依法创建的浓厚氛围，多年来难以解决的环境脏乱差、城市管理失序问题正在得到根治。群众满意度不断提升，城乡差距正在缩小，城乡融合发展取得新进展！

加大民生改善力度，人民群众幸福指数得到新提高。全年民生支出同比增长19.6%，占到一般公共预算支出的80.7%。多措并举促进就业创业，城镇新增就业超额完成省定任务。城乡低保人均标准、农村五保供养标准和老年人日间照料中心覆盖率保持全省领先。义务教育均衡发展，消除大班额和中考改革试点工作走在全省前列，阳泉职业技术学院新校区建成投用。医疗改革取得突破，公立医院薪酬制度改革走在全省前列，3个医联体覆盖了全市所有公立医疗机构。县乡医疗卫生机构一体化改革深入推进，平定县进入全省第一方阵。文化事业持续繁荣，第十三届三晋之春合唱比赛在我市成功举办，我市出品的现代晋剧《泥火情》公开演出，市博物馆完成布展并向公众开放。竞技体育再创佳绩，在第十五届省运会上我市奖牌总数位居全省前列，二青会射击射箭场馆建设积极推进。社区建设创出特色，城区被民政部命名为全国社区治理和服务创新实验区。城镇棚户区住房改造开工率、完成率排名全省第一。安全生产连续30个月杜绝较大事故，连续103个月杜绝重特大事故。扫黑除恶专项斗争深入推进，信访维稳走在全省前列，社会整体保持和谐稳定。十件民生实事全部落实。人民群众的获得感、幸福感、安全感不断增强！

持续转变政府职能，营商环境实现新改善。严格落实全面从严治党主体责任，坚持不懈推进党风廉政建设和反腐败斗争。提请市人大常委会审议地方性法规（草案）3件，出台政府性规章1件，办理人大代表建议218件、政协提案345件。“放管服效”改革进一步深化，全面推行企业投资项目承诺制，商事制度改革获国家市场监督管理局表彰，“双随机、一公开”监管工作走在全省前列。全市政务服务“一张网”基本实现，公共资源交易平台整合工作在全省率先完成，并实行全程电子化交易。“3545”改革取得突破，项目落地周期大大缩短。“阳泉随手拍”问政平台深得民心，“13710”督办系统高效运行。政府自身建设全面加强，行政效能不断提升！

与此同时，民族宗教、外事侨务、科普、档案、史志等工作取得新进展，妇女儿童、青少年、老龄、残疾人、红十字等事业有了新发展，国防动员、双拥、人民防空等各项工作呈现新气象。

各位代表，过去一年的成绩来之不易。这是以习近平同志为核心的党中央坚强领导的结果，是习近平新时代中国特色社会主义思想科学指引的结果，是全市上下在市委坚强领导下，团结一心、努力奋斗的结果！在此，我代表市人民政府，向全市人民，向各民主党派、工商联和无党派人士，向各位人大代表、政协委员，向驻地部队、公安民警和中央、省驻地单位，向所有关心支持阳泉发展的各界朋友，表示崇高的敬意和衷心的感谢！

在肯定成绩的同时，我们也清醒地认识到，我市发展仍面临不少困难和挑战，资源型老工业城市“一煤、一企、一股独大”的问题尚未得到根本性改变，结构性体制性素质性矛盾仍是经济发展的主要矛盾，支撑转型的项目、要素、环境都有较大差距；生态环保压力巨大，正处在爬坡过坎的关键时刻；改革红利尚未有效释放，开放水平有待继续提高，创新动力仍显不足，实体经济活力不够；政府职能转变还不到位，“放管服效”改革有待深入，营商环境还不理想；干部思想观念、能力素质与新形势新任务的要求还有不小差距，一些领域的不正之风和腐败问题不容忽视，等等。对此，我们必须强化底线思维，增强忧患意识，提高防范化解风险能力，充分估计困难，坚定必胜信念，做好打硬仗的准备，以更大的决心和意志，以更实的举措和行动，切实加以解决。

各位代表，当今世界面临百年未有之大变局，山西在“两转”基础上正在拓展新局面，我市处于转型发展的关键期和重大战略机遇期。我们一定牢牢把握机遇，积极应对挑战，保持战略定力，勇于担当作为，沿着高质量转型发展的道路坚定地走下去，奋力谱写新时代阳泉发展新篇章！

二、2019年工作安排

2019年是新中国成立70周年，是全面建成小康社会的关键之年，是我省在“两转”基础上拓展新局面的攻坚之年，是我市推动六大突破取得实质性进展的奋进之年。今年政府工作的总体要求是：以习近平新时代中国特色社会主义思想为指导，全面贯彻党的十九大和十九届二中、三中全会精神，统筹推进“五位一体”总体布局，协调推进“四个全面”战略布局，深入贯彻习近平总书记视察山西重要讲话精神，在市委坚强领导下，坚持稳中求进工作总基调，坚持新发展理念，坚持推动高质量发展，坚持“巩固、增强、提升、畅通”的八字方针，坚持省委转型发展“三条基本经验”，以供给侧结构性改革和转型综改试验区建设相结合为经济工作主线，围绕省委“三大目标”，按照市委聚力六大突破、实现转型崛起总体要求和“四个必须”基本要求，树牢“项目兴市”理念，在提升发展实力、增强市场活力、释放需求潜力上下功夫，全力做好“八项重点工作”，保持经济持续健康发展和社会大局稳定，为全面建成小康社会收官打下决定性基础，以优异成绩迎接中华人民共和国成立70周年。

2019年全市经济社会发展的主要预期指标是：全市地区生产总值增长6.3%左右，固定资产投资增长7.5%，社会消费品零售总额增长7.5%，一般公共预算收入增长3%，城镇和农村常住居民人均可支配收入分别增长6.5%和6.5%以上，城镇新增就业岗位2.1万个，城镇登记失业率控制在4.2%以内，居民消费价格涨幅控制在3%左右。约束性指标确保完成省定任务，并努力达到更好的目标。

围绕实现上述目标，今年重点抓好以下八个方面的工作：

（一）聚焦项目建设，夯实高质量发展的基础

2019年，全市计划实施315个项目，总投资1541亿元，年度计划投资274.3亿元。全年将以“百项重点工程”“五十个标杆项目”为引领，大抓项目，以项目促转型，以项目促改革，以项目促开放，真正把“项目兴市”的理念贯彻到项目工作的每个环节。

围绕能源革命抓项目。持续淘汰落后过剩产能，大力提升先进产能和新能源占比，走出一条资源型地区能源安全、清洁、绿色发展之路。煤炭方面，关闭和泰荫营煤矿，退出产能60万吨，推动阳煤西上庄500万吨、盂县坤宁120万吨煤矿开工建设。电力方面，加快裕光煤电盂县电厂2×100万千瓦项目建设，争取阳煤西上庄2×66万千瓦、盂县鑫磊2×35万千瓦低热值煤发电项目早日复工。新能源方面，加快中能建投平定、北京宣力李家山等总装机50.2万千瓦的13个风电项目建设，积极争取实施光伏领跑者计划二期项目，启动盂县2×1.5万千瓦生物质发电项目。加快煤层气开发利用步伐，推进二矿桑掌、盂县辰通、郊区神堂、固庄煤矿等煤层气发电项目。

围绕制造业振兴抓项目。将制造业振兴作为抓实体经济的根本，通过工业技改，以高端、智能、绿色为方向，全面提升制造业整体竞争力。现代煤化工方面，重点推进阳煤平定化工乙二醇二期项目，加快建设气凝胶、催化剂、化工创新中试基地等一批上下游相关产业项目，力争年内竣工投产一批。装备制造业方面，全力支持阳煤集团整合省内煤机制造企业，组建山西智能制造控股集团，鼓励阳煤华越机械、华鑫电气、奥伦胶带等企业开展技术创新，加快数字化改造，推动传统煤机由单一生产向全生命周期服务转变。非金属材料加工业方面，加快推进汉韵瓦业釉面瓦、鹏宝沃特陶瓷透水砖等项目建设，支持传统耐火、陶粒砂、陶瓷等向产业链高端延伸。节能环保产业方面，加快推进再生资源循环利用产业园建设，垃圾焚烧发电项目年内竣工投产，医疗废物、餐厨垃圾处理等项目加快建设进度；积极申报国家大宗工业固废综合利用示范基地，推动北新建材石膏面板项目落地开工。此外，加快推动河北生物医药等产业项目落户阳泉。

围绕基础设施建设抓项目。铁路方面，加快阳大铁路扫尾和综合交通客运枢纽建设，确保年底同步建成投运，抓紧市区石太货运铁路搬迁可行性研究。公路方面，加快国道307、207绕城改线工程进度，完成太行山一号旅游公路和“四好农村路”建设任务，加快推进国道239河底-荫营、国道207前洼-林里、省道盂榆线南上社-寿阳界等公路改线改建工程。市政道路方面，加快推进中环快速路北环贯通，完成平阳街二期东段、洪城北路北延、宁波北路、化义路等道路建设。水利方面，推进盂县乌河、平定南川河河道治理工程，启动城市应急备用水源龙华口调水工程前期工作，抓好郊区水务一体化项目，加快桃河、义井河河道综合整治。机场方面，加快推进盂县航空产业园项目建设。另外，抓住5G商用契机，加快布局信息化基础设施，助力数字经济发展。

围绕民生改善抓项目。继续实施冬季清洁取暖、采煤沉陷区综合治理、棚户区改造等工程，加快推进阳泉市养老公益苑、盂县安德森肿瘤医院等项目，特别是把市属国有企业“三供一业”移交后的63个维修改造项目与创卫工作结合起来，作为重点抓紧抓好。

围绕消费升级抓项目。着重发展与居民生活密切相关的交通、通讯、教育、医疗、金融、保险等服务业项目，推进消费结构向更高层次发展。拓展“互联网+消费”市场，积极培育电子商务、共享经济、信息消费等新业态。开工建设大型城市商业综合体，支持小米有品、乐村淘等企业布局网点，打造城市新零售模式。健全农村流通网络体系和售后服务体系，开拓农村消费市场。

围绕精准招商抓项目。编制转型产业规划和招商规划，创新以商招商、以资招商、乡情招商和股份制招商等方式，紧盯重点领域，重点在京津冀、长三角、粤港澳大湾区及我市进出口业务相对集中的“一带一路”国家举办务实有效的招商活动。开展好走访慰问阳泉籍在外工作人员“贴心行动”“迎老乡回故乡建家乡”等活动。对已签约项目强化跟踪服务，对有意向的大项目、好项目，盯住不放、快速跟进，确保落地见效。全市招商引资签约总额达到530亿元，当年签约开工率达到50%以上。

围绕服务保障抓项目。在项目融资、建设用地、环境容量、基础设施配套等要素方面给予转型项目更多倾斜。完善项目推进措施，强化市级领导包保责任、市直部门帮扶责任、县区政府直接责任和项目单位主体责任的“四级责任”，实行县区主要领导不定期调度、分管副市长逐月调度、市长双月调度、每季度向市委汇报的“四项机制”，扎实开展前期手续集中办理、项目集中开工、问题集中通办、要素集中集聚“四项活动”。一季度末，一半以上续建项目复工，三分之一以上新建项目开工；二季度末，续建项目全部复工，新建项目八成以上开工。

各位代表，高质量转型发展必须要有大项目、好项目支撑！我们将牢固树立“项目兴市”理念，把抓项目作为抓经济工作的第一要务，动员全市上下齐心协力抓项目，大干实干抓项目，为推动经济平稳健康发展提供更加有力的支撑！

（二）聚焦产业转型，培育高质量发展的新动能

推进传统产业改造提升和新兴产业培育壮大，努力构建多元支撑的现代产业体系。抓住供给侧结构性改革机遇，持续推动煤炭“减优绿”，进一步延伸煤炭产业链。深化电力体制改革，扩大直购电交易范围，推进增量配电网试点，扩展用电市场。抓住振兴制造业的机遇，夯实做强煤化、煤机两大基础制造业，培育壮大新材料、新能源等成长性制造业，推进先进制造业与现代服务业深度融合，提升制造业发展质量。抓住乡村振兴的战略机遇，加快发展有机旱作等现代特色高效农业和农业新产业新业态。抓住生态环保倒逼转型的机遇，加快发展节能环保产业。抓住山西获批国家通用航空业发展示范省的机遇，加快布局通用航空产业。

在新兴产业方面，重点发展四大产业。

抓住平台建设和场景应用两个关键环节，大力推进信息化，以信息化推动新一代信息技术产业发展。以建设国家新型智慧城市和智能物联网应用基地为契机，努力将我市建成全省新型智慧城市新标杆、智能物联网产业创新新高地。平台建设方面，完善政务云、基础数据库体系、共享交换等平台建设，做实信息化底层能力，为产业发展奠定基础。积极推进中国信通院智能物联网（阳泉）研究中心建设。深化与百度公司的合作，完成百度城市大脑核心能力平台建设，实现与共享交换数据平台的互联互通。推广应用方面，推动大数据、云计算、物联网、工业互联网在政务服务、机械制造、城市治理、交通物流、健康教育等领域的应用，加快推进在自动驾驶、智慧安防、智慧教育、智慧医疗、智慧社区、智慧旅游、AI城市展示平台等七大应用领域先行先试，打造AI城市品牌。以开发区各类双创平台为载体，大力发展轻资产、高附加值的大数据、物联网、人工智能等信息产业。

依托研发引进与改造提升两条路径，大力发展新材料产业。瞄准基础性、战略性、前沿性材料领域，积极引进新技术，研发新产品，培育壮大龙头企业，形成产业集群。以贝特瑞、西格里泉海为龙头，大力发展高端人造石墨、锂离子电池负极材料及高端新材料，形成具有较高成长性的新能源材料产业集群。以阳中新材为龙头，大力发展二氧化硅气凝胶全产业链新材料，力争三年内达到百亿级规模，形成具有自主知识产权的气凝胶产业集群。以中兴环能为龙头，大力发展洋葱碳、金刚石等纳米级炭材料。以长青公司等企业为龙头，通过技术创新，提升研发能力，形成具有核心竞争力的陶粒砂产业集群。加快推动铝矾土资源整合，鼓励耐火企业开展联合重组、技术升级、产品研发和数字化改造，把资源优势转化为产业优势，形成具有较强市场竞争力的耐火产业集群。

做实国际陆港和冠亚物流两大优势项目，大力发展现代物流业。积极主动参与区域物流产业分工，以国际陆港、冠亚物流等企业为龙头，加强与中国南山集团、天津物产集团等知名企业合作，打造具有保税、商检、海关等功能的现代化、国际化物流基地。积极推动天成煤炭集运站搬迁，并在原址建设阳泉综合保税物流中心（B型）。支持冠亚物流引进中国蓝田集团建设智能物流园，加快保税仓建设，力争年内投运。

坚持项目引领和文旅融合两个推动战略，大力发展文旅康养产业。加快全域旅游示范区建设，明确我市在长城太行旅游板块中的定位，突出太行特色，按“城景通、景景通”的要求，加快旅游公路及相关配套设施建设。深化与山西文旅集团的合作，按照国家5A级景区标准打造娘子关龙头景区。以大汖温泉康养小镇建设，带动一批康养类项目，积极引进战略合作者，鼓励一批特色旅游景区实现专业化运营。支持阳煤三矿工业遗址保护与开发。深度挖掘阳泉特色文化资源，开发民俗文化、民间工艺体验游产品。打造看民俗、住民宿特色旅游品牌。突出用好我市的名人效应，谋划打造科幻类文旅综合体验项目。加快智慧旅游建设，提升旅游服务质量。推进“文旅+”战略，打造独特的城市IP，努力凸显阳泉文旅的崭新风貌。

各位代表，转型任重道远，成功贵在坚持！我们将始终保持转型战略定力，以“功成不必在我、功成必定有我”的信念，横下一条心，培育新动能，尽快趟出一条资源型地区转型发展新路！

（三）聚焦关键领域改革，开辟高质量发展的新路径

深化国企国资改革，努力在僵尸企业出清、深度混改、国有资本运营上求突破。把今年作为我市国企国资改革攻坚之年，加快推进处僵治困，年内完成僵尸企业的破产程序、职工安置、社保接转等各项工作。扎实推进竞争类企业混合所有制改革，放开股权比例限制，扩大员工持股范围，鼓励各类资本参与，彻底解决国资一股独大、所有制结构单一问题。加快推进公共服务类、特殊功能类企业改革，形成规范有效的法人治理结构。积极引进战略投资者，推进市自来水公司特许经营改革，支持市煤气公司组建晋东燃气集团。在“三供一业”分离移交维修改造中，对水电气热进行智能化改造。积极盘活国有企业闲置土地、厂房、设备资源。完善国资管理体制，优化重组国有资本运营公司，年内具备融资功能。

深化开发区改革创新，努力在体制机制创新、平台打造、项目落地上求突破。对标山西综改示范区，构建精准高效适用的政策体系。推动平定经济技术开发区和阳煤集团管运分离改革取得实效，力争形成全省经验。支持盂县加快推进主体功能区规划调整，年内完成开发区设立批复。支持我市开发区与省内外先进开发区、大企业、科研院所、高校等合作共建产业园区。加强基础设施配套建设，积极引导社会资本参与开发区平台打造。加快阳泉经济技术开发区起步区建设，盘活闲置和低效利用土地。加快平定经济技术开发区2平方公里的平台打造和基础设施建设。探索标准地出让模式，做到“熟地等项目”。重中之重是发挥开发区在转型主战场的牵引作用，加大力度招引、建设项目，强化对开发区新建项目数量、土地有效利用率、投资强度、产出强度、税收强度等指标的考核，使开发区成为全市经济发展的“新引擎”。

深化财税金融体制改革，努力在强化绩效管理、保重点、防风险上求突破。全面实施预算绩效管理，提高财政资金使用效益。严格控制三公经费预算，取消低效无效支出，一律按照不低于5%的比例压减一般性支出。严格在省级核定限额内申请发行并合理安排地方政府债券，适当向标杆项目和重点工程倾斜。严格规范PPP建设项目、政府购买服务等行为，有序化解隐性债务存量，坚决遏制增量。按照国、省部署，推进税制改革。深化金融体制改革，加快平定、郊区农信社改制步伐，加大不良贷款处置力度，规范阳泉市商业银行发展。与阳煤集团共同设立“双阳”产业转型母基金。全市应急周转保障资金规模扩大至2亿元，各县区不少于2000万元。加强地方金融监管，严打严防经济犯罪，筑牢金融安全堤坝。

深化农村集体产权制度改革，努力在规范“三资”管理、深化股份改革、做强集体经济上下功夫。进一步完善农村集体资产清产核资和集体经济组织成员身份确认，规范农村集体“三资”管理。开展集体资产股份合作制改革，将经营性资产折股量化到人、确权到户，推动资源变资产、资金变股金、农民变股东，真正让农民享受到改革红利。建立新型农村集体经济组织，不断发展和壮大集体经济。建立健全农村产权交易市场，完善运行机制，提升服务水平，实现农村产权入市公开交易。全面完成农村集体产权制度全国试点任务，力争在全国形成经验。

各位代表，改革是决定转型成败的关键一招，阳泉的改革决不能落后！我们将继续坚持在创新中求突破，打破体制机制藩篱，真正使改革成为转型发展的源头活水！

（四）聚焦补短板强弱项，激发高质量发展的新活力

全力做大做强民营经济。坚持“两个毫不动摇”，落实我省支持民营经济“30条”和我市“1+6”行动方案。进一步放宽民营经济市场准入，取消不合理的限制规定。支持“泉民投”公司发挥作用，鼓励民间资本参与国企混改和政府投资项目建设。依法保护民营企业家合法权益，认真清理政府部门和国有企业拖欠民营企业账款。积极帮助民营企业争取专项资金扶持，鼓励金融机构加大信贷支持力度，用好中小微企业信保基金，着力破解民营企业融资难融资贵问题。构建亲清新型政商关系，完善领导干部联系民营企业制度，健全企业家参与涉企政策制定机制。推进小微企业上规升级，今年新培育“小升规”企业8家、“专精特新”企业4家。

不断提升科技创新能力。以创建国家创新型城市为抓手，进一步完善依靠科技创新推动转型升级的政策体系，组织实施30个以上重大自主创新和产业化项目，全面启动创新型县区建设工作，力争平定获批省级创新型县区。推进产学研深度融合，支持企业与科研院所、高等院校等开展合作。重点培育100家科技型中小企业、65家高新技术企业，打造30家市级及以上重点实验室和工程技术中心。支持双创示范基地建设，搭建“6+1”特色双创平台，支持大唐369云工厂等创新平台提档升级。建立科技创新双创学院，推动建立区域孵化联盟。建立柔性人才引进机制，充分发挥中国信通院智能物联网（阳泉）研究中心的人才支撑作用，加强院士工作站建设，加大科技领军人才引进力度。继续开展“晋商晋才回乡创新创业工程”。积极实施全市青年拔尖人才支持计划，赋予人才发展更多自主权。

切实提高对外开放水平。加快融入“一带一路”大商圈，主动对接京津冀、长三角、大湾区等国家战略，落实好国家支持山西与京津冀加强协作实现联动发展的意见，加强与北京跨区域产业协作中心的合作，在北京非首都功能疏解和雄安新区建设中寻求机遇，承接产业梯度转移。加快对外开放平台建设，建设综合保税物流中心（B型）和区域铁路物流集散中心。充分发挥阳泉海关职能优势，复制自贸区改革经验，最大限度实现投资与贸易便利化。支持跨境电商外贸综合服务平台等新业态发展。支持外贸企业做大做强，开拓海外市场。放宽市场准入，积极引进外商投资项目。加大本地产品出口推介力度，探索在境外联合办展和自办展模式。加强友城合作，扩大海外朋友圈。

各位代表，发展的高度取决于短板的长度!我们将坚持问题导向，瞄准差距所在，紧盯痛点、难点和堵点，精准发力，靶向施策，让高质量发展的活力充分迸发！

（五）聚焦城乡融合，城乡并举促进区域协调发展

高起点规划设计蓝图。按照“一核多极”总体布局，合理规划生产、生活、生态空间，打造南到平定、北到荫营的中心城区，加快建设西烟镇、孙家庄镇、张庄镇、娘子关镇等特色小镇和一批中心村，形成特色鲜明、层次分明的城乡融合发展格局。启动“多规合一”编制和生态新城起步区城市设计。积极推进行政区划调整工作，解决“城矿郊”规模结构不合理问题。

高标准建设中心城区。按照“北扩南提东进西融”发展思路，推动城市空间南北延伸、东西拓展。老城原则上多拆少建，完善基础设施，改善城市风貌，年内完成德胜街、天成巷、巨兴街、阳泉西高速路口、207国道五渡至大西庄城市风貌改造工程，推进城市公园绿化和片区绿地改造。新城建设力求有所突破，加快推进新城骨干路网建设，城市规划展览馆、射击射箭馆投入使用，开工建设AI智慧公园，积极推进阳泉市中心医院、阳泉大剧院建设，让新城成为阳泉城市新名片。

高质量推进乡村振兴。不断深化农业供给侧结构性改革，实施城郊农业发展工程，大力发展设施蔬菜、畜牧养殖、干鲜果、花卉苗木等高效农业，积极推进高标准农田建设，新建6个标准化种植示范基地，打造1000亩杂粮标准化生产示范区。实施农产品加工业提升工程，培育壮大农业龙头企业，加快发展“互联网+农业”。实施休闲农业与乡村旅游推进工程，促进农林文旅康产业融合发展。实施品牌农业创建工程，培育区域公用品牌。实施有机旱作农业发展工程，建设300亩以上有机旱作农业示范点10个。实施新型经营主体培育工程，积极培育农民专业合作社联合体和示范家庭农场、合作社、农业产业化联合体。落实好社会资本“上山下乡”的支持性政策，鼓励人才、政策、资金和更多的工商资本、社会资本投资农业农村。

高水平推进城乡环境综合整治。统筹开展“五城联创”，决战决胜创卫攻坚，大力开展环境综合整治、市容市貌综合整治、健康食品卫生提升三大行动，重点是新建和提标公厕、新建改造垃圾中转站，持续整治城市“十乱”、农贸市场、便民市场和早夜市、第五立面和“六小行业”，完善“多勤合一”长效管理机制，继续深化环卫市场化外包服务改革，打造阳泉创卫特色，力争全面达标通过验收。今年市县联动推进创卫工作，同步改善城乡人居环境。学习浙江“千村示范、万村整治”经验，农村实施拆违治乱、垃圾治理、污水治理、厕所革命、卫生乡村五大专项行动。加快美丽宜居乡村建设，新启动3个示范片、20个示范村、100个提升村的整治工作。扎实做好“大棚房”问题专项清理整治，毫不松懈抓好非洲猪瘟防控。

高效能巩固提升脱贫成效。在整市脱贫的基础上，围绕“两不愁三保障”目标，坚持摘帽不摘责任、不摘政策、不摘帮扶、不摘监管，落实好后续扶持各项工作，建立返贫预警机制，设立防贫保险险种。研究制定针对收入水平略高于建档立卡贫困户群体的支持政策。支持已脱贫村开展美丽宜居创建活动，打造10个脱贫奔小康示范村、10个休闲农业和乡村旅游脱贫成果巩固提升示范村。

各位代表，城乡融合发展是社会发展的必然要求，是乡村振兴的基本路径！我们将拆除城乡之间的藩篱，实现生态环境共治、基础设施相通、公共服务共享、生产要素在城乡之间顺畅流动，让城乡之间的差距越来越小！

（六）聚焦营商环境优化，为高质量发展提供基础保障

深化“放管服效”改革，在便民化上见实效。引深审批制度改革，积极探索“一枚印章管审批”的相对集中行政许可权改革，加快政务服务标准化建设，持续开展“减证便民”行动。推行“承诺制+并联审批”模式，推进“承诺制+标准地”改革，试点区域评估，实行告知承诺、容缺受理制度，压缩核准类项目审批评估时限。深化“3545”专项改革，大幅压减企业开办、不动产登记、建设项目审批及水电气报装办理时限。深化商事制度改革，全面推行证照分离，大力推进照后减证，推进企业登记全程电子化，最大限度让企业用营业执照一把钥匙，打开“准入”和“准营”两扇大门。创新事中事后监管方式，全面构建以“双随机、一公开”监管为基本手段、以重点监管为补充、以信用监管为基础的监管机制，推进跨部门联合监管和“互联网+监管”，对新兴产业实施包容审慎监管。健全守信联合激励和失信联合惩戒机制。放开中介市场准入，依法整治红顶中介。优化政务服务，加快推进“马上办、网上办、就近办、一次办”全覆盖，新建市级政务服务大厅投入使用，推动政务服务事项应进必进，构建一窗受理、集成服务模式。“12345”政务服务热线平台建成投用。完善政务服务体系，加快推进数据共享、一网通办，到年底实现政务服务平台市县乡村四级全覆盖，80%以上事项可以网上办理，必须到现场办的事项力争做到“只进一扇门、最多跑一次”。

落实各项惠企政策，在降成本上见实效。积极落实国家和省已出台的减税降费政策，继续阶段性降低企业社保费率。提升互联网办税水平，大幅压减企业办税时间。全面推行依清单收费，坚决整治乱收费、乱罚款。完善工业用地弹性出让制度，加快“公转铁”运输结构调整，降低企业要素成本。优化企业银行开户服务，规范涉企审批程序，压缩审批时限，降低企业制度性交易成本。

创新管理体制机制，在营造公平竞争环境上见实效。建立公平竞争审查制度，有序清理妨碍统一市场和公平竞争的各类显性和隐性障碍，打破行政性垄断，防止市场垄断。建立完善市场准入负面清单制度，推动“非禁即入”普遍落实。按照全省统一部署，积极配合做好营商环境评价评估工作，以评促改、以评促优，营造公平公正的法治环境、规范守信的市场环境、重商亲商的社会环境。

各位代表，“水深则鱼悦，城强则贾兴”，项目的建设、企业的壮大、人才的引进、活力的激发，一刻也离不开良好的营商环境！我们将把优化营商环境作为关键性工作大力推进，紧盯“六最”目标，以营商环境的明显改善为高质量发展提供坚强保障！

（七）聚焦生态环保攻坚，治理修复双管齐下见实效

坚决打赢蓝天保卫战。这是我们对人民的庄严承诺，我们将坚持“五管齐下”，以超常举措尽快扭转生态环保不利局面。持续调整优化产业、能源、运输、用地结构，坚持排放总量与排放标准双控制，标准服从总量。严格实行工业企业错峰生产，持续推进重点行业特别排放限值改造，杜绝散乱污企业死灰复燃。开展建筑工地绿色施工，加强道路扬尘综合整治，煤场料场、渣土车全封闭，加快推进矸石山治理，到2020年实现退役矸山治理全覆盖。持续开展柴油货车和散装物料运输车污染治理联合执法。加快燃煤锅炉淘汰和整治，完成煤改电、煤改气和集中供热6.6万户。提升大气污染热点网格平台服务能力，加强空气质量监测，科学研判、分级预警、及时应对重污染天气。严格落实党政同责、一岗双责和“管发展、管生产、管行业必须管环保”的要求，强化督察执法，严格考核问责，倒逼责任落实。

持续打好碧水保卫战。统筹推进“五水同治”，深入开展集中式饮用水水源地专项行动，全面整治县级及以上水源保护区内违法违规问题。加快城镇生活污水处理厂提效改造，推进污水管网雨污分流改造，确保污泥处置设施建成投运。推进桃河、义井河、南川河、香河、秀水河等污染严重河段环境综合整治工程，巩固黑臭水体治理成效。深度处置工业水，确保煤矿外排矿井水达到地表水三类标准。结合农村人居环境改善，确保农村生活污水乱排乱放得到有效管控。

统筹打好净土保卫战。完成农用地土壤污染状况详查，公布污染地块清单，强化土壤污染源监管，加强农业面源污染防控，加大对城市周边裸地、历史遗留矿坑点治理力度。

抓好生态修复和体制机制建设。深入开展国土绿化行动，完成各类造林7.51万亩。推进自然资源统一确权登记，开展自然资源资产负债表编制工作。突出抓好环境监测、生态环境损害赔偿、排污许可、区域环评等重点改革任务。强化国土空间规划和“三线一单”管控。深入推进环保机构监测监察执法垂直管理体制改革，健全乡镇街道环保机构，将环保监管延伸到最基层。

各位代表，绿色发展已经成为新时代的最强音，发展再不能以牺牲环境为代价！我们将增强忧患意识，坚守生态底线，以更加坚毅的决心和自觉的行动，奋力爬坡过坎，开辟转型路径，真正让绿色成为美丽阳泉的底色！

（八）聚焦社会民生，着力提高人民群众幸福指数

千方百计稳定和扩大就业。进一步加大就业创业支持力度，紧盯高校毕业生、去产能安置职工、农村劳动力、就业困难人员、退役军人等重点群体，构建精准就业帮扶机制，确保零就业家庭动态清零。支持困难企业开展职工在岗培训，鼓励企业不裁员或少裁员，稳定劳动关系。加快人力资源市场建设，加强全方位公共就业服务。全年完成城镇失业人员再就业1.3万人，转移农村劳动力0.8万人。

持续提升社会保障水平。继续实施全民参保计划，稳步提高各项社会保险待遇水平。落实城乡居民基本养老保险待遇确定和基础养老金正常调整机制，做好城乡低保和特困供养提标工作。全面放开养老市场，推进养老机构公建民营。建立健全覆盖城乡的多层次医疗保障体系。推进殡葬改革，加快市殡仪馆搬迁。关爱农村特殊群体，大力发展妇女、儿童、老龄、慈善和红十字会等事业。完善住房市场体系和保障体系，推进城中村改造和非集中成片棚户区改造，推进农村危房改造，开工建设1100套保障房，实现“居者有其屋”。

大力发展文化教育事业。扩大普惠性学前教育资源。优化义务教育资源配置。探索中小学教师“县管校聘”改革和校长职级制改革。推进中考改革试点工作。改善高中办学条件。促进职业教育提档升级，支持阳泉职业技术学院和阳泉师专建设发展优势专业。加快构建现代公共文化服务体系，推进基层综合文化服务中心建设。加强优秀文化遗产保护传承，推进文明守望工程、革命文物保护利用工程和乡村文化记忆工程。继续开展各类文化惠民工程。繁荣文艺创作，开展文化交流。

提升全民健康水平。深化县乡医疗卫生机构一体化改革，推动城区、矿区分别以市一院和阳煤总院为龙头探索市域医联体建设。深化公立医院综合改革，巩固完善薪酬分配制度，探索院长聘任制。持续推进医疗、医保、医药“三医联动”，更好保障群众就医需求。推进“三特兴医”工程，打造一流医院和领军专科。推进医疗信息化建设，完善分级诊疗、双向转诊和远程医疗。

保持社会安全稳定。牢固树立安全发展理念，提高风险预知预警预判能力，紧盯重大敏感节点，严防群体性事件、突发环境事件、重大食品药品安全事故、重大交通事故等公共安全事件。压实党政、部门、企业安全责任，全面构建风险分级管控和隐患排查治理双重预防机制，深化重点领域专项整治，坚决杜绝重特大事故，有效遏制较大事故。强化食品安全工作责任制，保障人民群众“舌尖上的安全”。高压严打私挖滥采和盗采国家矿产资源行为。加强和创新社会治理，完善社会矛盾纠纷多元化解机制，妥善做好各类群体的利益诉求信访工作。发挥工会、共青团、妇联等群团组织作用，推动社会治理重心下移。做好民族宗教、援疆工作。加强双拥和退役军人服务管理，支持国防建设。加强气象、地震、人防等工作，构建应急管理体制机制，做好防灾减灾救灾工作。持续推进扫黑除恶专项斗争，切实维护社会和谐稳定与人民群众生命财产安全，守好山西东大门。

各位代表，今年我市将承办第二届全国青年运动会的射箭比赛。我们将全力以赴，高质量完成场馆建设，打造出优美的城市环境，精心做好服务保障工作，充分展示阳泉的美好形象。

各位代表，为政之要，在于为民！今年，市政府将在去年的基础上，重点办好10件民生实事：继续实施全民技能提升工程；为1878名残疾人实施残疾预防重点干预和残疾儿童抢救性康复；继续为怀孕妇女提供免费产前筛查与诊断服务；新建23个农村老年人日间照料中心；免费送戏下乡550场；实施经济困难的高龄和失能老年人关爱行动工程；实施免费法律咨询便民工程；提高城乡居民基本医疗保险财政补助标准；主城区新建5个公共停车场，1100个停车位；新建改建130座公厕，改造6600座农村户厕。我们就是要把改善民生、惠及百姓的实事一件一件办成，一件一件办好，不断满足人民群众对美好生活的期待！

三、以“改革创新、奋发有为”大讨论为牵引，全面加强政府自身建设

各位代表，开启新征程，完成新目标，必须进一步加强政府自身建设。我们将积极推进政府机构改革，尽快实现人员到位、职责到位、工作到位，切实改进工作作风，加快转变政府职能，全面提高行政效能。

（一）以“改革创新、奋发有为”大讨论牵引全年工作。从2月中旬到4月底，省委部署开展“改革创新、奋发有为”大讨论，这是省委十一届七次全会作出的重大决策，是我省贯彻落实习近平总书记关于改革开放再出发和新时代新担当新作为指示精神的重大举措，对推动阳泉改革创新、思想解放意义重大。山西大发展，阳泉要先行！我们将按照市委安排，以大讨论牵引全年工作开局，着力破除僵化保守、因循守旧、封闭狭隘、资源依赖、随遇而安、慵懒散漫等6方面问题，坚持领导带头，突出分类指导，抓好统筹融合，自我对标对表，扎实推进12个规定动作，迅速兴起大讨论热潮，推动大讨论成为思想觉醒、革故鼎新，求真务实、拓展新局，守住底线、勇攀高峰的学习实践，共同凝聚追梦奋斗、振兴崛起的强大正能量。

（二）坚持以政治建设统领全面工作。把党的政治建设摆在首位，进一步增强“四个意识”，坚定“四个自信”，做到“两个维护”，坚决贯彻落实党中央、国务院大政方针和省委省政府、市委决策部署。强化理论学习，持续深入开展政府系统能力提升大讲堂学习，推动学习成果转化为工作思路和工作举措。严肃开展党内政治生活，模范遵守党章党规，不断提高科学民主决策水平。深化廉政风险防控，加强重点领域监督管理，努力实现干部清正、政府清廉、政治清明。

（三）坚持以法治方式推进工作。严格执行人大及其常委会的决议决定，认真办理人大建议、政协提案，自觉接受民主监督，强化审计监督。建立健全行政规范性文件管理制度和合法性审核机制，依法办理行政复议和行政应诉案件。全面推进政务公开和政府信息公开。

（四）坚持以优良作风提升效能。严格落实中央八项规定，持续纠正“四风”，坚决反对形式主义、官僚主义。充分发挥“13710”督办平台作用，及时解决“阳泉随手拍”反映问题，确保政策落地、政令畅通。完善干部考核评价机制和容错纠错机制，大力整治不担当、不作为现象，激发改革创新热情，保护干事创业积极性，确保政府工作人员勤勉尽责，努力当好人民公仆。

各位代表，机遇与挑战并存，动力与压力同在！让我们更加紧密地团结在以习近平同志为核心的党中央周围，高举习近平新时代中国特色社会主义思想伟大旗帜，在市委的坚强领导下，紧紧依靠全市人民，砥砺奋进，勇于担当，扎实工作，奋力开拓转型发展新局面，以优异成绩迎接中华人民共和国成立70周年！
